# Attempts at Veterinary Colleges

**Published:** 1883-01

**Authors:** 


ATTEMPTS AT VETERINARY COLLEGES.—We announced in our last that a Veterinary Department was to be established in connection with the Harvard Medical School. It turns out that the Veterinary Department consists at present of only one Professor.

If this means that it is only a beginning, very well. But if it is expected to create veterinarians by means of one Professor's teaching, we strongly protest.

It is quite impossible to become practical and skillful in comparative medicine by studying in a human medical college. We gladly welcome the appearance of new veterinary colleges, but the attachment of veterinary Professors, however skillful, upon the faculty of a medical college for the sake of picking up stray students, is a practice to be condemned.

